# The Linkage Among Gut Microbiota, Inflammatory Cytokines, and Immune Cell Dynamics in Osteoporosis: A Mendelian Randomization Study

**DOI:** 10.33549/physiolres.935563

**Published:** 2025-10-01

**Authors:** Yuan GUO, Jinghua DU, Hui CHEN

**Affiliations:** 1 Department of Medical Laboratory, Northern Jiangsu People’s Hospital Affiliated to Yangzhou University, Yangzhou City, Jiangsu Province, China; 2 Department of Medical Laboratory, Northern Jiangsu People’s Hospital, Jiangsu Province, China; 3 Department of Endocrinology, Northern Jiangsu People’s Hospital Affiliated to Yangzhou University, Yangzhou City, Jiangsu Province, China; 4 Department of Science and Technology, Northern Jiangsu People’s Hospital Affiliated to Yangzhou University, Yangzhou City, Jiangsu Province, China; 5 Department of Science and Technology, Northern Jiangsu People’s Hospital, Jiangsu Province, China

**Keywords:** Gut Microbiota, Inflammatory cytokines, Immune cell dynamics, Osteoporosis, Mendelian randomization

## Abstract

This investigation attempted to discern the causal link of gut microbiota with osteoporosis, examining potential mediating factors, involving inflammatory markers and immune cell activity. Bidirectional two-sample univariable Mendelian randomization (UVMR) was used to decipher the causal link of gut microbiota with osteoporosis, verifying three core assumptions. External datasets were utilized to validate UVMR outcomes and implemented reverse analyses to identify potential reverse causality. Additionally, mediation effects were figured out through UVMR, estimating effect sizes and proportions for every qualifying mediator. It was attempted to precisely select instrumental variables (IVs), ensuring that those influenced by linkage disequilibrium (LD) or demonstrating weak correlations were excluded. The inverse-variance weighted (IVW) analysis unveiled 12 gut microbiota species that were remarkably linked with osteoporosis risk. Specifically, five families, involving *Pasteurellaceae*, could elevate the risk of osteoporosis, while another five, such as *Oxalobacteraceae*, were protective. Additionally, two inflammatory markers exhibited a remarkable linkage with osteoporosis following heterogeneity testing, and 37 distinct immune cell types were recognized as being relevant to the disease after adjusting for heterogeneity and pleiotropy. Reverse MR analysis confirmed the absence of reverse causality among gut microbiota, inflammatory factors, immune cells, and osteoporosis. Notably, mediation analysis unveiled that *Cyanobacteria* influenced HLA DR^++^ monocytes’ percentage in leukocytes, contributing to osteoporosis’s pathogenesis. The outcomes highlighted specific gut microbiota, inflammatory factors, and immune cells, noticeably contributing to osteoporosis’s pathogenesis. The identified mediating pathways provided innovative insights into disease mechanisms and potential therapeutic targets.

## Introduction

As a highly prevalent skeletal disorder, osteoporosis is defined by a noticeable decrement in bone mass and the progressive degradation of bone microarchitecture, culminating in an elevated susceptibility to fractures. The global burden of osteoporosis is profound, with an estimated 200 million cases affected worldwide, resulting in substantial morbidity, mortality, and healthcare expenditures arising from osteoporotic fractures. Although postmenopausal women are disproportionately affected, osteoporosis also poses a notable health threat to older men, where it remains markedly underdiagnosed and undertreated [1]. In light of the ongoing demographic shift toward an aging universal population, osteoporosis’ incidence is steadily rising. Consequently, comprehending the pathophysiology of osteoporosis, as well as advancing strategies for its prevention and management, has become an urgent focus of medical research and public health, particularly in aging populations.

Emerging evidence has highlighted a potential, yet still incompletely understood, link of the gut microbiota with bone health. The gut microbiota may influence bone density through a range of mechanisms, involving the modulation of systemic inflammation, the regulation of nutrient absorption, and regulating hormonal pathways that may impact bone metabolism [2,3]. Notably, certain microbial taxa have exhibited to correlate with variations in bone mineral density (BMD) across different populations, providing compelling evidence for the microbiome’s involvement in bone health [4]. However, the causal link of gut microbiota composition with osteoporosis remains elusive, and the precise molecular mechanisms of this association require additional investigation. Dysbiosis, or microbial imbalance, in the gut microbiota has been implicated in osteoporosis’s pathogenesis through its effects on systemic inflammation and calcium homeostasis, both of which are critical determinants of bone integrity. Manipulation of the gut microbiota, *via* interventions, such as antibiotic use, dietary modifications, and supplementation with prebiotics and probiotics, has exhibited to have remarkable impact on bone health [5]. Specific microbial communities, involving *O. Burkholderiales* and *G. Ruminococcus*, have been implicated in the modulation of bone metabolism through the microbiota-gut-bone axis, suggesting that microbial composition may have a direct function in osteoporosis’s development and progression. Experimental studies, particularly those conducted on ovariectomized mice, have further elucidated the function of the gut microbiota in bone loss, indicating that the microbiome may impact bone health through both immune and metabolic pathways [6]. A particularly intriguing direction of research involves targeting the bile acid receptor TGR5, which has exhibited to be involved in microbiota-driven bone loss, presenting a potential therapeutic target for mitigating postmenopausal osteoporosis [7].

Traditional observational research is typically constrained by confounding variables and the potential for reverse causality, obscuring the true nature of the linkage of gut microbiota with osteoporosis. Mendelian Randomization (MR), however, is accompanied by a more robust and methodologically sound alternative by leveraging genetic variants regarded as instrumental variables (IVs) to decipher causal links. This technique effectively diminishes the restrictions of confounding and reverse causation, providing more accurate insights into causal mechanisms [8]. MR emerged in form of a powerful tool in epidemiological research, having successfully elucidated causal links in numerous complex traits and diseases. By emulating the conditions of a randomized controlled trial (RCT), MR enables the establishment of causality of risk factors with disease outcomes in a manner that is less susceptible to biases inherent in traditional observational designs. Mediation analysis, often incorporated within MR frameworks, further enhances causal inference by evaluating how an exposure exerts its effects on an outcome through an intermediary variable or mediator [9].

A growing body of MR research has figured out recently the intricate links of gut microbiota with osteoporosis, inflammatory mediators and bone density, immune cell dynamics and bone metabolism, as well as the interconnections among gut microbiota, plasma metabolites, and osteoporosis [10–15]. For instance, MR research has concluded a positive link of interleukin-7 (IL-7) levels with osteoporosis, while other interleukins have exhibited no noticeable linkage with the disease [16]. In addition, elevated levels of IL-27 have been recognized as a risk factor particularly for osteoporosis, with evidence suggesting a causal link of IL-27 with the disease [17]. Further investigations have highlighted the critical role of circulating metabolites with inflammatory mediators in the pathogenesis of postmenopausal osteoporosis (PMOP), with a notable causal link of certain plasma metabolites with PMOP [18]. In this context, IL-16 has been regarded as a mediator that links inflammatory signaling to osteoporosis-related effects. The role of inflammatory cytokines, particularly TNF-α, alongside immune cell populations (e.g., T cells), in regulating both bone resorption and bone formation has been well documented, confirming their notable involvement in osteoporosis’s pathophysiology [19]. Despite these outstanding advancements, there remains a paucity of comprehensive research that examines the complex, bidirectional interactions among gut micro-biota, inflammatory factors, and immune cells within the context of osteoporosis.

This study was precisely designed to decipher the causal linkage of gut microbiota with osteoporosis, utilizing the sophisticated MR analytical framework. By leveraging genetic variants that serve as reliable IVs for both gut microbiota composition and osteoporosis susceptibility, it was attempted to unravel the intricate causal pathways that link these two domains. The goal was not only to elucidate the direct microbial influences on bone health, but also to identify specific microbial taxa whose modulation may hold promise as novel therapeutic targets for osteoporosis. In addition, through the application of MR, the mediating function of the gut microbiota in shaping the linkage of genetic predisposition with osteoporosis risk was examined, thereby deepening comprehending of how microbial composition interacts with genetic factors to influence bone health outcomes. A remarkable component of this investigation was to decipher the potential mediating influence of inflammatory markers and immune cell dynamics within the gut microbiota-bone axis. By incorporating these inflammatory and immune factors into the causal model, the complex link among the microbiome, immune system modulation, and bone metabolism, along with novel mechanistic insights into osteoporosis pathogenesis, could be uncovered.

In summary, this investigation attempted to provide a comprehensive and multidimensional understanding of the function of gut microbiota in the initiation and progression of osteoporosis. Through the advanced application of MR techniques, the limitations of traditional observational studies were eliminated, enabling us to generate causal inferences that could revolutionize the prevention and therapeutic management of osteoporosis.

## Materials and Methods

### Study design and methods

**Fig. 1:**
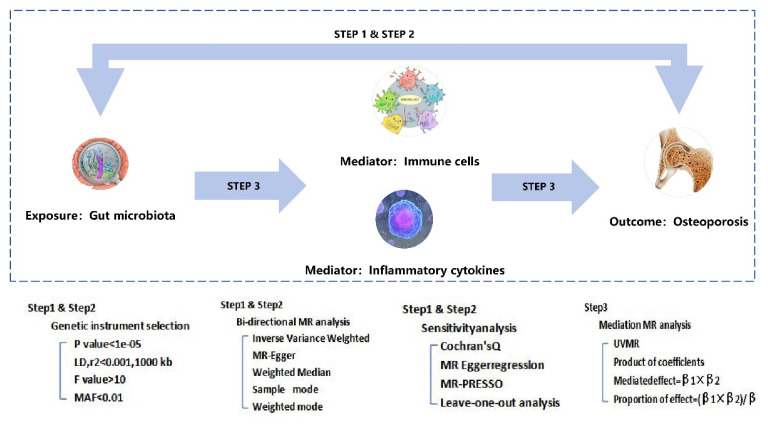
Schematic displaying the Mendelian randomization (MR) study.

The methodological framework of this MR investigation was systematically executed in three distinct phases (Fig. 1). During the first two phases, a bidirectional two-sample univariable MR (UVMR) approach was implemented to rigorously interrogate the causal link of exposure with outcome. This approach hinged on three fundamental assumptions: (1) the selected single nucleotide polymorphisms (SNPs) had a robust linkage with the exposure variable, (2) the SNPs influenced the outcome exclusively *via* the exposure, ensuring specificity of the causal pathway, and (3) they were independent of any confounding factors. A reverse causality assessment was also integrated to discern potential bidirectional causal influences, with external datasets utilized to validate the UVMR outcomes’ robustness. In the third phase, a concurrent UVMR mediation analysis was implemented to quantify the mediation effects particularly between exposure and outcome, computing both effect sizes and proportional mediation contributions for every qualified mediator. The study especially adhered rigorously to the STROBE-MR guidelines, thereby ensuring comprehensive reporting standards for epidemiological observational studies.

### Data sources

The characteristics of the GWAS data sources employed in this investigation are accessible in Table S1. Summary data on gut microbiome (GM) from the MiBioGen consortium involved a comprehensive analysis of genome-wide genotypes and 16S fecal microbiome profiles for 18340 cases drawn from diverse ancestries across 24 distinct cohorts, with European participants constituting 78 % of the sample population. To ensure robust identification of microbiome quantitative trait loci (mbQTLs), only taxa detected in over 10 % of samples were retained, yielding a dataset that encompasses 211 taxa across various taxonomic ranks, comprising 131 genera, 35 families, 20 orders, 16 classes, and 9 phyla. This stringent selection enabled precise mapping of genetic loci linked to GM abundance, thereby refining the comprehending of host-microbiome genetic interplay at multiple taxonomic levels [20].

The GWAS Catalog, comprising entries GCST90274758 through GCST90274848, represents a perfect repository for genome-wide association statistics on circulating inflammatory proteins, encompassing data for 91 inflammatory cytokines derived from a sample of 14,824 cases of European ancestry [21]. Moreover, details on these 91 inflammatory markers are accessible in Table S2.

In this investigation, mediation analyses were implemented leveraging GWAS summary statistics on both inflammatory cytokines and immune cell phenotypes. Data concerning immune cell phenotype were drawn from 3757 cases of European descent across non-overlapping cohorts, covering a broad spectrum of 731 immune-related features: relative cell counts (RC, n=192), absolute cell counts (AC, n=118), cellular morphological parameters (MP, n=32), and median fluorescence intensities indicative of surface antigen expression (SAL and MFI, n=389) [22]. These phenotypic features involved mature immune cell stages, specifically panels of B cells, regulatory T cells (Tregs), monocytes, T cells, CDCs, TBNK subsets (B cells, natural killer cells, T cells), and myeloid cells. MP features concentrated on parameters pertinent to CDC and TBNK panels. The comprehensive list of all 731 immune cell traits is cataloged in Table S3.

To ensure a robust selection of osteoporosis-related data from the Finnish biobank resource, cohort size, study publication date, SNP quantity, and participant ancestry were rigorously evaluated. The finalized datasets consist of 399,054 cases of European descent, involving 8017 osteoporosis cases and 391037 controls (Table S1) [23].

### Selection of genetic IVs

The genetic IV selection protocol followed a comprehensive multistep approach: initially, to enhance SNP efficacy, a significance threshold of 1e-05 was applied to IVs linked to GM, IC, IF, and OS. Next, SNPs in linkage disequilibrium (LD) with r2<0.001 within a 10000 kb span were systematically excluded. Subsequently, SNPs that displayed significant linkage with the outcome (p<5e-05) were also removed to avoid confounding. Palindromic SNPs were further excluded to maintain alignment of allelic directionality across exposure and outcome associations. Additionally, the F-statistic was employed to unveil IV robustness, retaining only SNPs with F-statistics exceeding 10 (SNPs with minor allele frequency (MAF) below 0.01 were removed) (Supplementary Tables).

### MR analyses

It was attempted to undertake MR analyses through a suite of methodological approaches, involving weighted mode, inverse-variance weighted (IVW), weighted median, MR-Egger, and simple mode. Serving as the primary MR analysis tool, the IVW method aggregates the Wald ratios of individual SNPs *via* meta-analytic techniques, assuming that IVs impact outcomes solely through the exposure pathway. This assumption is critical for obtaining unbiased causal estimates, highlighting the absence of horizontal pleiotropy. Thus, the IVW approach was central in deriving robust causal inference in the current investigation. To deepen the analytical rigor and mitigate potential distortions arising from invalid IVs or horizontal pleiotropy, utilization of MR-Egger methods and the weighted median was implemented. These strategies, however, are sensitive to outlier genetic variants, with MR-Egger particularly vulnerable, possibly reducing precision. The weighted median approach is accompanied by reduced bias yet compromises some accuracy. Although less efficient, weighted median, simple mode, MR-Egger, and weighted mode were involved as supplemental methods to substantiate the outcomes [24–27]. This research complies with STROBE-MR guidelines, as confirmed by the checklist.

### Mediation MR analysis

Potential mediators in the gut microbiota-osteoporosis pathway were systematically screened through a multi-step process, as outlined in Figure 1. In the primary stage, UVMR was employed to recognize mediators that were causally influenced by the exposure, and their respective effect sizes (denoted as b1) were computed. In the secondary stage, UVMR was again applied to choose the mediators discerned in the primary stage that causally impacted the outcome, followed by determining their causal effect sizes (denoted as a). The third stage involved ensuring logical consistency in the effect directions: if exposure’s total effect on the outcome (b) was positive, both the exposure-mediator effect (b1) and the mediator-outcome effect (a) were expected to exhibit either positive or negative signs, respectively; however, if exposure’s total effect particularly on the outcome (b) was negative, b1 and a were expected to differ in sign. In the final step, multivariable MR (MVMR) was conducted to assess the causal effect of mediators on the outcome while adjusting for the effect of the exposure. Mediators exhibiting a MV-IVW P-value inferior 0.05 were regarded as the final candidates for inclusion.

Next, the causal effect of exposure on the outcome (b) was assessed, derived from the UVMR analysis, and employed the “product of coefficients” method to compute the mediated effect values for every potential mediator within the gut microbiota-osteoporosis pathway, expressed as (b1×b2). It was attempted to determine the proportion of mediation by computing the ratio of the mediated effect value to the total effect ([b1×b2]/b).

In the two-sample MR (TSMR) analysis (steps 1A and 2A in Fig. 1), gut microbiota, inflammatory cytokines, and immune cells were incorporated with a significant causal linkage to osteoporosis as potential mediators in the pathway. The causal link of gut microbiota with cytokines/immune cells was figured out in step 3 (path a, Fig. 1). If a causal association was confirmed, additional MR analyses were executed to examine whether cytokines and immune cells could function as intermediaries in the pathway from gut microbiota to osteoporosis. The significance of the mediated effects was quantified using the Sobel test, involving computation of the P-value through the online platform.

### Sensitivity analysis

A comprehensive sensitivity analysis was implemented to scrutinize potential sources of heterogeneity and pleiotropy. To this end, both IVW and MR-Egger regression methodologies were applied, with Cochran’s Q statistic computed to quantify the extent of heterogeneity across the involved IVs. In parallel, the intercept term from the MR-Egger regression was assessed to decipher the presence of horizontal pleiotropy, which may distort causal inference. It was attempted to execute statistical analyses through R 4.4.1 software, and implementation of MR analyses was through the “TwoSampleMR” package [28]. Additionally, to enhance the analysis’ efficiency, certain computational procedures were executed *via* the fastMR package.

## Results

### Positive MR

Notably, 12 distinct types of gut microbiota were identified to be associated with osteoporosis risk, of which two were excluded due to significant heterogeneity. Among the remaining 10, 5 taxa were associated with an increased risk of osteoporosis, while the other 5 were associated with a decreased risk.

**Fig. 2:**
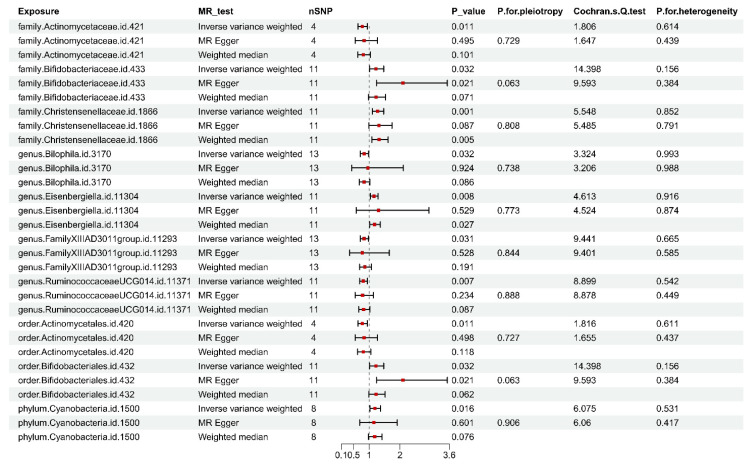
Outcomes from the Mendelian Randomization (MR) analysis examining the linkage of gut microbiota with osteoporosis risk.

By employing a TSMR framework, ten robust associations were delineated between gut microbiota and osteoporosis (FDR>0.1, P_IVW<0.05). Notably, the Bifidobacteriaceae family, along with the genera Bifidobacterium and Eisenbergiella, the order Bifidobacteriales, and the phylum Cyanobacteria, exhibited a positive association with increased susceptibility to osteoporosis. In contrast, the genus Bilophila, the Actinomycetaceae family, the order Actinomycetales, the genus Ruminococcaceae UCG014 (classified within the Ruminococcaceae family), and the Family XIII AD3011 group showed inverse associations with osteoporosis risk, suggesting potential protective roles. These associations are further elaborated in Figure 2 and Table S4. To decipher the MR findings’ robustness and reliability, sensitivity analysis was conducted, as illuminated in Figure 2 and Table S5. Cochran’s Q test was executed, and the absence of significant heterogeneity was noteworthy among the IVs, reinforcing the validity of the associations. Additionally, MR-Egger regression analysis provided no indication of horizontal pleiotropy, further substantiating the causal links inferred in the current investigation. A comprehensive summary of the TSMR analysis figuring out the influences of GM on OP is accessible in Table S6.

**Fig. 3:**
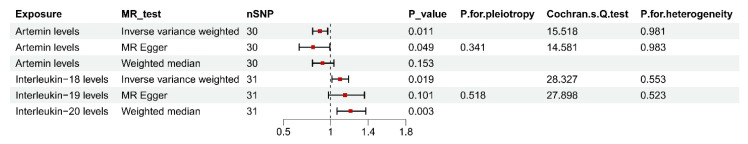
Outcomes of Mendelian randomization analysis of inflammatory factors linked with osteoporosis.

This investigation identified a notable link of three inflammatory factors with osteoporosis. These included IL-18, IL-19, and Artemin. Upon conducting a heterogeneity assessment, IL-19 was excluded from subsequent analysis due to significant heterogeneity, while the remaining two factors, IL-18 and Artemin, demonstrated remarkable links with osteoporosis. Specifically, escalated levels of IL-18 were positively linked with an elevated risk of osteoporosis, confirming a potential pro-inflammatory function in its pathogenesis. In contrast, higher concentrations of Artemin were inversely linked with osteoporosis risk, indicating a protective effect. For a comprehensive presentation of these associations, Figure 3 is noteworthy, and further outcomes are outlined in Tables S7–9.

**Fig. 4:**
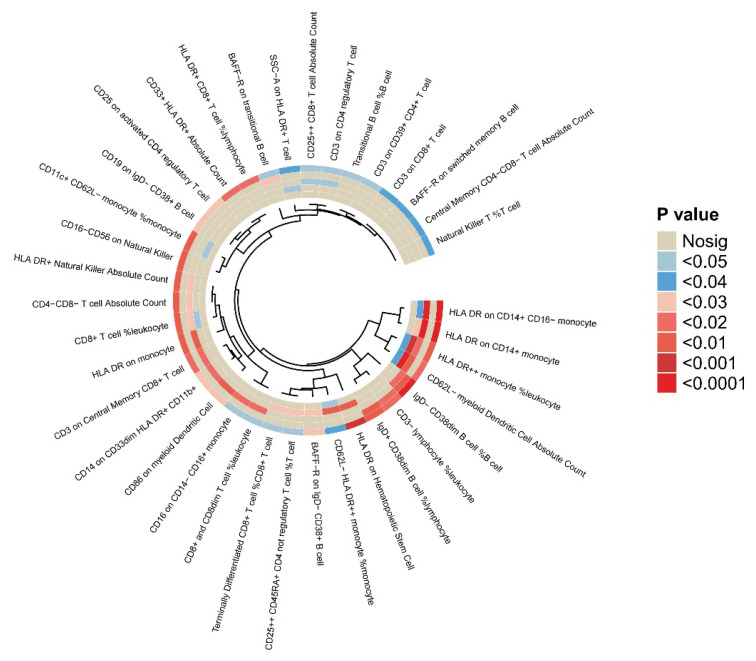
Outcomes of Mendelian randomization analysis of immune cells linked with osteoporosis.

This comprehensive analysis identified 47 immune cell traits associated with osteoporosis risk. After excluding 10 traits based on multifactorial analysis and heterogeneity assessment, 37 immune-related traits demonstrated robust associations. Among those linked to a reduced risk of osteoporosis were increased percentages of transitional B cells, higher proportions of CD11c^+^ CD62L^−^ monocytes, elevated BAFF-R expression in IgD^−^ CD38^+^ B cells, increased CD25 expression in activated CD4^+^ regulatory T cells, and higher counts of HLA-DR^+^ natural killer (NK) cells. Additional protective associations included increased abundance of central memory CD8^+^ T cells, elevated counts of CD4^−^CD8^−^ T cells – representing a subset of unconventional T cells with potential immunoregulatory roles – enhanced BAFF-R expression in both transitional and switched memory B cells, a higher proportion of IgD^+^ CD38^dim B cells among lymphocytes, and increased frequency of CD25^++^ CD45RA^+^ CD4^+^ non-regulatory T cells. Conversely, traits associated with an increased risk of osteoporosis included higher counts of CD33^+^ HLA-DR^+^ myeloid cells, elevated percentages of NKT cells among T cells, increased HLA-DR expression in hematopoietic stem cells and monocytes, and elevated CD14 expression in CD33^dim HLA-DR^+^ CD11b^+^ cells. Further associations with increased risk involved greater abundance of CD25^++^ CD8^+^ T cells, higher proportions of IgD^−^ CD38^dim B cells and CD3^−^ lymphocytes among leukocytes, increased counts of CD62L^−^ myeloid dendritic cells and CD4^−^CD8^−^ T cells, as well as elevated CD19 expression in IgD^−^ CD38^+^ B cells. Moreover, increased CD86 expression in myeloid dendritic cells and higher percentages of CD8^+^ and CD8^dim T cells among leukocytes were also observed in association with elevated osteoporosis risk. These findings collectively suggest that a range of both innate and adaptive immune cell populations may influence osteoporosis susceptibility through diverse immunoregulatory mechanisms. Further details are available in Figure 4 and Table S10–12.

### Reverse MR

Osteoporosis was regarded as the exposure variable, whereas gut microbiota, inflammatory factors, and immune cells were considered as outcome variables. As shown in Table S13–15, the absence of reverse causal associations among gut microbiota, inflammatory factors, immune cells, and osteoporosis is particularly noteworthy.

### Mediating effect

The findings revealed that both the gut microbiome and cytokines exert causal influences on osteoporosis. However, no direct causal links were identified between osteoporosis-associated gut microbiota and the cytokines involved in the condition, indicating that cytokines did not mediate the relationship between gut microbiota and osteoporosis. Further details are presented in Table S16–17.

In the forward MR analysis figuring out the influences of gut microbiota on 37 immune cell types implicated in osteoporosis, four remarkable links were identified. Notably, the phylum Cyanobacteria (id.1500) exhibited a noticeable influence on the SSC-A of HLA-DR^+^ T cells (P=0.035, β=0.247, se=0.117). Similarly, the order Actinomycetales (id.420) could impact HLA-DR^+^ CD8+ T cells’ percentage among lymphocytes (P=0.034, β=−0.331, se=0.156). In parallel, the family Actinomycetaceae (id.421) also influenced HLA-DR^+^ CD8^+^ T cells’ percentage in lymphocytes (P=0.035, β=−0.330, se=0.156). Additionally, the phylum Cyanobacteria (id.1500) demonstrated a remarkable linkage with HLA-DR^++^ monocytes’ percentage particularly in leukocytes (P=0.004, β=−0.318, se=0.111). Crucially, a mediating effect was noteworthy involving the gut microbiome phylum Cyanobacteria (id.1500), with linkage with osteoporosis, and immune cells characterized by HLA-DR^++^ monocytes’ percentage in leukocytes, which are similarly linked with osteoporosis. This outcome uncovers that cytokines may function as mediators in the pathway linking gut microbiota to osteoporosis. For a comprehensive review of these results, Table 1 and Table S18–20 are worthy of study.

## Discussion

Recent investigations have elucidated the intricate linkage of gut microbiota with osteoporosis [29], affirming findings of the present study. For instance, MR analysis unveiled ten specific taxa in gut microbiota as influential in osteoporosis pathogenesis: five taxa linked with an augmented risk, while five appeared inversely related, underscoring the highly multifaceted etiology of osteoporosis [13]. This finding parallels this study’s observations regarding particular gut microbiota families, involving *Pasteurellaceae* and *Oxalobacteraceae*, which exhibited positive and negative links with osteoporosis risk, respectively.

Moreover, a comprehensive narrative review has emphasized gut microbiota’s regulatory function in bone metabolism, proposing that the depletion of microbial diversity may exacerbate both osteoporosis and osteoarthritis [30]. This corroborates this study’s findings, demonstrating that distinct microbiota taxa exert influence on bone health, potentially through immune-modulating pathways. Similarly, further investigations have posited that restructuring gut microbial communities *via* fecal microbiota transplantation supports the concept of a “gut-bone axis” [31].

In addition, research on alcohol-induced osteoporosis in a rodent model has highlighted the bidirectional links of gut microbiota composition with immune regulation. Prolonged alcohol exposure not only altered the gut microbiome, but also intensified osteoporotic progression, particularly in aging subjects [32]. This phenomenon aligns with this study’s outcomes that unveiled a remarkable link of 37 distinct immune cell types with osteoporosis, suggesting that gut microbiota may modulate bone health through immune cell dynamics and inflammatory processes.

Complementary insights arise from a study investigating postmenopausal osteoporosis, which discerned robust linkage of the *Burkholderiales* order with both heightened osteoclast activity and a mitigated risk of osteoporosis. This research further isolated primary genes, such as *FMNL2* and *SRBD1*, appearing to underlie these associations [33]. These findings resonate with the identification of gut microbiota-mediated impacts on immune cells and inflammatory pathways, aligning with the identification of gut microbiota-mediated impacts on immune cells and inflammatory pathways.

In contrast, alternative studies provided disparate perspectives. For instance, research concentrating on the dietary modulation of the gut microbiota has pointed out that nutritional patterns may influence osteoporosis *via* the gut-bone axis, emphasizing the function of dietary intake in shaping gut microbial composition and, subsequently, bone metabolism [34]. Although the present investigation did not figure out dietary factors, the foundational mechanisms involving gut microbiota’s impact on bone health remain congruent with this study’s findings.

Gut microbiota may be indispensable in mediating bone metabolism and the progression of osteoporosis. The present research aligns with studies that delineated the complex interrelationships among gut microbiota, inflammatory mediators, and immune cells in relation to bone health. The gut-bone axis has exhibited as a remarkable function through which microbiota modulate bone density and architectural integrity. Certain microbial families, such as *Pasteurellaceae*, have exhibited linkage with an escalated osteoporosis risk, whereas others (e.g., *Oxalobacteraceae*) appeared to confer protective effects against bone degradation [13].

Inflammatory mediators have a central and multifaceted function in osteoporosis’s pathophysiology, as evidenced by the identification of specific inflammatory cytokines linking with alterations in BMD. Chronic systemic inflammation could exacerbate osteoclastogenesis, thereby promoting bone resorption by activating osteoclasts as the fundamental effector cells responsible for bone degradation. The inflammatory markers discerned in the current investigation resonate with previous research that underscores the notable function of systemic inflammation in the progression of osteoporosis [30].

The linkage of immune cells, particularly various subsets of T lymphocytes and monocytes, with bone homeostasis introduces an additional layer of complexity to the gut microbiota-bone health axis. Immune cells could modulate bone remodeling through the secretion of cytokines and other molecular mediators, directly impacting osteoclast activity and, consequently, bone resorption. A pivotal study has unveiled 13 novel immune phenotypes that exhibited a causal linkage with osteoporosis [12], thereby providing a conceptual framework for the emerging field of bone immunology. The present study’s findings confirmed notable links of specific immune cell subsets with osteoporosis risk. Furthermore, the identification of HLA DR^++^ monocytes as mediators in the gut microbiota-bone health pathway may provide novel mechanistic insights into how immune cells may influence bone metabolism, particularly in the context of gut microbiota perturbations.

The reverse MR analysis adds a crucial dimension to this discourse, revealing that osteoporosis does not significantly alter gut microbiota composition, inflammatory markers, or immune cell profiles. This unidirectional link unveils that while gut microbiota and immune system factors can influence bone health, the progression of osteoporosis itself does not appear to reciprocally affect these biological systems.

Furthermore, this investigation highlighted *Cyanobacteria* as a pivotal microbial taxon that could mediate the linkage of gut microbiota with bone health by influencing the activity of HLA DR^++^ monocytes. This discovery underscores the potential of specific microbial species in modulating immune responses that have downstream effects on bone health. This microbial-immune interaction may help clarify how specific taxa influence immune function relevant to bone health [35,36].

Consequently, this investigation documented compelling evidence of the complex linkage among gut microbiota, inflammatory mediators, and immune cells in the context of pathogenesis of osteoporosis. The identification of specific microbial families, inflammatory markers, and immune cell subsets linked to osteoporosis boosts comprehending of the disease’s molecular and immunological underpinnings.

Through bidirectional TSMR, the current investigation provided a more robust and notable approach to decipher causality than previous research, which has predominantly concentrated on the link of gut microbiota with bone health [13]. The present study’s findings corroborated earlier studies demonstrating that gut microbiota could influence bone metabolism through immune and inflammatory mechanisms [30,32], while this knowledge was extended by identifying specific microbial families, such as *Pasteurellaceae* and *Oxalobacteraceae*, that are robustly linked with osteoporosis risk. This level of specificity enhances comprehending of the gut-bone axis [37].

Furthermore, the integration of MR to decipher the mediating functions of inflammatory factors and immune cells introduces a sophisticated layer of complexity to explore the causal pathways in osteoporosis. This methodological approach was further validated utilizing recent studies that concentrated on the multifactorial nature of osteoporosis, involving not only host genetic factors, but also microbial and immune system interactions [32]. The present study’s findings are in harmony with prior research that highlighted the noticeable function of specific inflammatory markers and immune cell subsets in preserving bone homeostasis, thus reinforcing the robustness and credibility of this study’s analytical framework [30,34].

The reverse MR analysis implemented in this investigation, which revealed no significant reciprocal influence of osteoporosis on gut microbiota composition, inflammatory mediators, or immune cell profiles, provides strong support to the hypothesis of a unidirectional link of gut microbiota with bone health. This is a critical distinction, effectively mitigating concerns regarding reverse causality, which is a typical limitation inherent in observational studies. By leveraging external datasets for validation, the scientific integrity of this study’s findings was further upgraded, ensuring their generalizability and enhancing the study design. This comprehensive methodological approach contributes substantially to advance comprehending of the gut-bone axis in osteoporosis research.

In conclusion, by integrating robust MR methods and mediation analysis, this study revealed specific microbial families, immune cell subsets, and inflammatory markers causally linked to osteoporosis. These findings enhance the mechanistic understanding of the gut-bone axis and may inform future directions for therapeutic modulation of gut microbiota or immune responses in the context of osteoporosis.

## Limitations

Nonetheless, several limitations merit consideration. Firstly, no wet laboratory experiments were implemented, which would provide a direct, empirical basis for the molecular and cellular mechanisms identified. Secondly, while the sample size was regarded sufficient for robust statistical analysis, while it may still be regarded as relatively modest in the context of capturing the full spectrum of gut microbiome diversity and its noticeable influences on osteoporosis. Thirdly, the absence of clinical validation in real-world settings remains a remarkable gap, as these findings require confirmation through clinical trials and patient-based research. Additionally, the usage of multiple datasets might introduce the potential for batch effects, which, despite rigorous adjustments, could influence the outcomes. Furthermore, since this cohort was predominantly of European descent, the generalizability of the findings to non-European populations remains uncertain, necessitating further investigation into more ethnically diverse cohorts.

## Conclusions

In summary, the current investigation has delineated critical microbial mediators that could impact osteoporosis’ pathogenesis. Through the usage of both UVMR and MVMR, a causal link between gut microbiota and osteoporosis has been established, alongside a quantification of the mediation effects of specific microbial factors. These outcomes highlight the indispensable function of the gut microbiome in bone health and set the stage for future research aimed at advancing microbiome-targeted therapeutic interventions for osteoporosis. Moving forward, future studies will prioritize the validation of the outcomes through clinical trials and experimental models, alongside wet laboratory investigations to provide mechanistic insights, ultimately facilitating the translation of these discoveries into clinical strategies for osteoporosis management.

## Supplementary Information









































## Figures and Tables

**Table 1:** Mediation analysis of the effect of gut microbiota on osteoporosis *via* immune cells.

Exposure	Mediator	Outcome	Total effect	Direct effect	Mediation effect (95 %CI)	*P*-value	Mediation Proportion
phylum.Cyanobac-teria.id.1500	HLA DR^++^ monocyte %leukocyte	Osteoporosis	0.211	0.173	0.079 (0.008, 0.079)	0.032	37.44 %

## Abbreviations

BMD: bone mineral density
GM: gut microbiome
IVs: instrumental variables
IVW: inverse-variance weighted
LD: linkage disequilibrium
MR: Mendelian Randomization
RCT: randomized controlled trial
SNPs: single nucleotide polymorphisms
UVMR: univariable Mendelian randomization
